# Strengthening Community-University Engagement at Illinois to Support Aging and Disability

**DOI:** 10.1093/geroni/igaf122.3170

**Published:** 2025-12-31

**Authors:** Carrie Wennerdahl, Laura Rice, Wendy Rogers

**Affiliations:** University of Illinois Urbana-Champaign, Champaign, Illinois, United States; University of Illinois College of Applied Health Sciences, Urbana, Illinois, United States; University of Illinois Urbana-Champaign, Champaign, Illinois, United States

## Abstract

University and community engagement creates meaningful and mutually beneficial partnerships that enriches learning experiences and provides a foundation for research that is relevant and impactful; addresses community needs; builds trust; and enhances the validity and application of the findings. At the University of Illinois Urbana-Champaign, the College of Applied Health Sciences has two research themes: CHART (Collaboration on Health, Aging, Research, and Technology) and CARD (Collaborations in the Advancement of Research on Disability). Together, they represent consortia of faculty across campus and are proactively developing community connections with local leaders, healthcare providers, community agencies, older adults, people with disabilities, and caregivers. We will demonstrate how CHART and CARD have played a critical role in fostering community and university partnerships with successful programs and events this past year, as part of our Age-Friendly University mission. We hosted an open house and tour of the McKechnie Family LIFE Home during Active Aging Week to showcase research innovations with smart home technologies and robotics for people of all ages and abilities. We conducted a symposium entitled “Community/University Engagement to Support Aging and Disability” with over 100 attendees that brought together community members and researchers to explore strategies for effective research partnerships. Presentations and a panel discussion highlighted the importance of collaboration and addressing the needs of diverse communities. These initiatives not only strengthen university-community connections but also pave the way for continued collaboration and implementation of research initiatives to promote meaningful change and enhance well-being of individuals across all generations and abilities.

